# Assessing genetic diversity and similarity of 435 KPC-carrying plasmids

**DOI:** 10.1038/s41598-019-47758-5

**Published:** 2019-08-02

**Authors:** Christian Brandt, Adrian Viehweger, Abhijeet Singh, Mathias W. Pletz, Daniel Wibberg, Jörn Kalinowski, Sandrina Lerch, Bettina Müller, Oliwia Makarewicz

**Affiliations:** 1 Institute for Infectious Diseases and Infection Control, Jena University Hospital, Jena, Germany; 2 BioCenter, Department of Molecular Sciences, Swedish University of Agricultural Sciences, Uppsala, Sweden; 3 Institute of Medical Microbiology, University Hospital, Leipzig, Germany; 4 Bioinformatics Faculty of Mathematics and Computer Science, Friedrich Schiller University, Jena, Germany; 5 InfectoGnostics Research Campus, Jena, Germany; 6 Center for Biotechnology, Bielefeld University, Bielefeld, Germany

**Keywords:** Computational biology and bioinformatics, Clinical microbiology

## Abstract

The global spread and diversification of multidrug-resistant Gram-negative (MRGN) bacteria poses major challenges to healthcare. In particular, carbapenem-resistant *Klebsiella pneumoniae* strains have been frequently identified in infections and hospital-wide outbreaks. The most frequently underlying resistance gene (*bla*_KPC_) has been spreading over the last decade in the health care setting. *bla*_KPC_ seems to have rapidly diversified and has been found in various species and on different plasmid types. To review the progress and dynamics of this diversification, all currently available KPC plasmids in the NCBI database were analysed in this work. Plasmids were grouped into 257 different representative KPC plasmids, of which 79.4% could be clearly assigned to incompatibility (Inc) group or groups. In almost half of all representative plasmids, the KPC gene is located on Tn*4401* variants, emphasizing the importance of this transposon type for the transmission of KPC genes to other plasmids. The transposons also seem to be responsible for the occurrence of altered or uncommon fused plasmid types probably due to incomplete transposition. Moreover, many KPC plasmids contain genes that encode proteins promoting recombinant processes and mutagenesis; in consequence accelerating the diversification of KPC genes and other colocalized resistance genes.

## Introduction

The global spread and diversification of multidrug-resistant Gram-negative (MRGN) bacteria with plasmid-localized β-lactamases poses major challenges to healthcare. β-lactamases in general are ancient enzymes^1^ and most likely developed in the environment^2^. The transmission to pathogenic bacteria might result from increasing industrial antimicrobial pollution of natural habitats^3^. It has been demonstrated that sublethal concentrations promote stable, high-level resistance at low fitness costs^4^. Highly selective urban and hospital environments promote the diversification and exchange of resistance genes, particularly on conjugable or mobilizable plasmids, which are additionally associated with transposons^5^. In particular, carbapenem-resistant *Klebsiella pneumoniae* strains have been frequently associated with infections and hospital-wide outbreaks^6,7^. The most frequent underlying resistance gene, *bla*_KPC_, is mainly located on a plasmid. KPC-2 and KPC-3 are predominantly identified (32 alleles are stored at NCBI BioProject 313047 as of January 2019)^8^.

KPC has been reported to be often associated with so-called pkpQIL-like plasmids, mostly due to an infamous nationwide outbreak with highly carbapenem-resistant *K*. *pneumoniae* in Israel in 2006, which led to a detailed sequence analysis of this plasmid^9^. Furthermore, researchers in other countries, such as the USA^10^, the UK^11^, Greece^12^, Italy^12^ and Taiwan^13^ have identified pkpQIL plasmids. In addition to pkpQIL-plasmids, many other KPC-bearing plasmid types have been identified^14–16^. The localization of KPC-β-lactamases on a transposon seems to be mainly responsible for different KPC-plasmids, with Tn*4401* being the most prominent transposon type for KPC-2^17^. Moreover, KPC-carrying plasmids often contain other genes for antibiotic resistance, virulence or detoxification as well as genes related to plasmid stability and longevity^10,11^.

The result of this transposon-directed transfer can continuously lead to different rearranged and newly mixed KPC plasmid types and thus to novelty. The aim of this study was to illustrate the result of this transposon-directed transfer by analysing the distribution of KPC and its association with different plasmid backbones.

The characterization of these KPC plasmids is of particular interest not only for outbreak control, but also for epidemiological surveillance of antibiotic resistance, because resistance plasmids of Gram-negatives usually carry multiple resistance genes and are exchanged between species^18^.

In order to avoid an overestimation of frequently described and deposited plasmids (e.g. pKpQIL type), a comparison based only on representative sequences was needed. However, similarity matching or clustering utilizing more conserved sequences (e.g., such as genomic DNA) could not be used because there is no consistent core genome between plasmid types. While a conserved ‘mosaic-like’ backbone exists within a plasmid type^19,20^ they cannot be easily utilized for a general overview across all KPC plasmid types. Furthermore, it is unclear how to deal with the mixed core backbone in fusion plasmids or plasmids with Inc-colocalization to achieve a reliable bioinformatic characterization of plasmid types.

To analyse the genetic heterogeneity of all KPC plasmids currently available at NCBI, a plasmid clustering approach tailored to this problem was applied.

## Results and Discussion

### Plasmid clustering into representative sequences

In total, we identified 759 *bla*_*KPC*_-bearing sequences in the NCBI database (May 2019). After selecting only circular plasmid sequence entries, 435 *bla*_KPC_-bearing plasmid sequences with sizes ranging from 7,995 bp (Accession Number KC609322) to 447,095 bp (Accession Number CP029436) were investigated.

Because plasmids usually contain a variable number of transposons and insertion sequences, they cannot be grouped properly with conventional genome analysis or binning tools (e.g. based on gene presence or absence). Furthermore, there is no consistent ‘core genome’ or a universal typing method that can be applied across different types of plasmids^21^. Moreover, mobile genetic elements can be large and could make up a significant proportion of the plasmids, creating close phylogenetic relation if a distance-based method is applied. As such, we used a clustering-based approach via psi-cd-hit.pl (detailed description in the Method section) to separate the plasmids into 257 representative KPC-plasmids (from here simply referred to as ‘representatives’). The clustering results can be found in the Supplementary Material Table S1, Section 2. We adjusted the psi-cd-hit.pl setting to consider certain plasmid properties for this large set of plasmid types. For example, many genes are similar for different plasmid types due to selective advantages (e.g. conjugation, toxin/antitoxin systems, antibiotic resistance). Therefore, only high scoring pairs (HSPs) covering 10% of the query were accepted, to avoid spurious clustering based solely on a similar gene content. This means that an HSP contains a minimal gene arrangement due to its length. In addition, the total length of all HSPs must cover at least 60% of the longer sequence. This setting was used to consider the occurrence of fusion plasmids. Many smaller KPC plasmids below 100 kbp would otherwise not be considered, because they match to fusion plasmids of 200 kbp or more. Only the representative sequence of each cluster (as labelled via psi-cd-hit.pl) was used for further analysis.

However, some limitations must be noted. Assembling plasmids remains difficult, especially for short reads and large amounts of repetitive sequences, as found in transposon or insertion sequences. This can lead to unfinished plasmid contigs or uncommon plasmid chimeras due to an incorrectly resolved assembly graph, which can be usually resolved with long-read sequencing technologies^22^. In our case, 63.4% of all 257 representatives (retrieved from GenBank) were sequenced using either PacBio (Pacific Biosciences) or Nanopore (Oxford Nanopore Technologies) technology, which should be assembled to current standards (e.g. hybrid assembly via Unicycler^23^; if long and short reads are available). Only circular sequences entries were used (as specified in the GenBank record), which should remove most incomplete sequences. Furthermore, only the representative sequence of each cluster is investigated. Plasmids with only a few missing genes (e.g. by deletion, gene loss or incomplete) would still be clustered to the representative sequence. Since most of the data available for KPC-plasmids were related to clinical samples, there was a bias favouring clinical pathogens.

**Figure 1:**
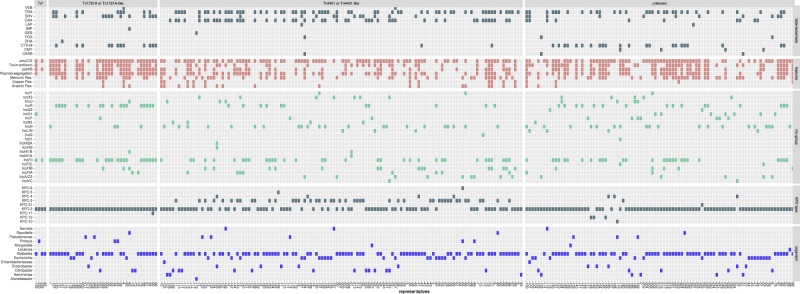
Schematic presentation of the gene content and organism distribution for each representative KPC plasmid ordered by the identified transposon element. Each column corresponds to a representative plasmid. Each column is numbered based on the entry ‘representative number’ in the Supplementary Table S1, Section 1. *Enterobacteriaceae* corresponds to unclassified *Enterobacteriaceae bacterium*.

All representatives were used to analyse genes of interest such as β-lactamases, toxin systems or other elements such as Inc groups and transposon regions. We arranged the representative plasmids based on the identified transposon types and summarized the extracted information in Fig. 1 (detailed results for each representative plasmid are given in Supplementary Material Table S1, Section 1). The species and the isolation date were extracted only from the representative plasmids. Information on microbial origins of other plasmid members of a cluster was not considered.

### Incompatibility groups

**Figure 2:**
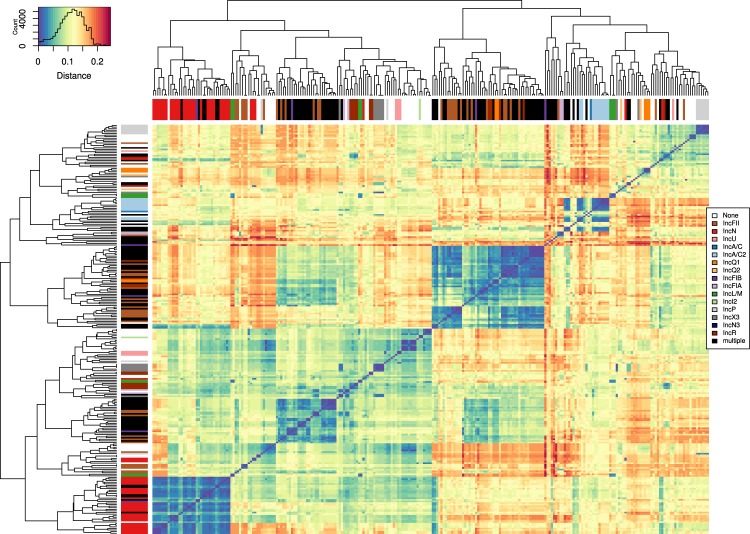
Heatmap diagram of the distances between the individual 257 representative plasmids plotted using the heatmap.2 function in R. The blue/red gradient represents the estimated Mash distance (legend at the top left, reflects the amount of data corresponding to each colour/distance). Dark blue indicates closely related representatives with similar gene content but different gene arrangements. On the X and Y axes, the 257 representatives were arranged based on their distance to each other, and the branches are annotated with colours that correspond to the different plasmid incompatibility groups (legend on the right).

In total, we identified Inc groups in 204 representatives using the PlasmidFinder database^24^. 128 out of 257 (49.8%) representatives could be assigned to a single specific Inc group (Fig. 2), with IncN (n = 30) being the most prevalent group, followed by IncFII (n = 29), IncR (n = 11) and IncA/C2 (n = 10). Multiple Inc group colocalizations were identified in 76 out of 257 (29.6%) representatives, with IncFII & IncR (n = 25) and IncFIB & IncFII (n = 12) occurring most frequently (for more details see Supplementary Material Table S1, Section 1).

The similarity among all 257 representatives was assessed by estimating the distance between them using Mash^25^ (Fig. 2, detailed heat map in Supplementary Material Fig. S1). In order to identify representative plasmids, minimal gene ordering was required for clustering (see section ‘Plasmid clustering into representative sequences’), due to minimal HSP size. Thus, a small distance now reflects either (a) similar gene content but different order or (b) that representative sequence is partially or fully represented in a larger fusion/hybrid plasmid. Although the distance-based approach cannot be used to group representative plasmids in a tree like representation (or generally different types of plasmids), it provides an overview of the sequence content similarity between the representative plasmids as a heatmap. For the sake of clarity, the representatives were sorted based on distance in the figure. These sorted groups still show high similarities to various other distinct groups, due to the previously raised points about plasmid types with shared genes or mobile elements. The heatmap was useful to illustrate the behaviour of overall plasmid similarity between different Inc groups and Inc colocalizations.

### Transposons

We identified in 165 of the 257 representatives the following transposon types Tn*4401*-like (n = 124), Tn*1721*-like (n = 37) and Tn*6367*-like (n = 4). Only matches with a minimal sequence coverage of 60% were considered. In ten representatives, the same transposon type (Tn*4401*-like or Tn*1721*-like) was present twice (representative number 7, 15, 23, 26, 95, 111, 113, 134, 160 and 190, detailed coverage and hit information in the Supplementary Material Table S1, Section 1). The majority (n = 72) of the remaining 92 representatives contained several transposon-related genes (retrieved from GenBank entries), such as *tnpR* or *tnpA*, which were mainly associated with Tn*3* or Tn*3*-like structures, suggesting the presence of other active transposons. Transposon-specific genes did not appear to be restricted to a particular Inc group.

The high transposon frequency in the representative plasmids indicates that the often-described transposon-mediated transposition of KPC-β-lactamases remains the primary mechanism for the spread of the *bla*_KPC_ gene to other plasmids. Especially Tn*4401* or its variants were identified in 48.2% (124/257) of all representatives. The oldest described KPC-2 found in *K*. *pneumoniae* that was isolated in 1997 and was located on an IncN-plasmid within the Tn*4401b* transposon^26,27^. Tn*4401* is a Tn*3*-class transposon that can mobilize *bla*_KPC-2_ genes under laboratory conditions at a frequency of 4.4 × 10^−6^ per recipient cell^28^. Tn*1721A*-like structures carrying *bla*_KPC_ were identified in representative plasmids originating from Japan, Taiwan and China^29^. Tn*1721A*-like transposons seems to be preferable located on IncFII/IncR colocalized KPC-plasmids (12 of 37 representatives).

### Resistance gene alleles

The KPC-2 was the most abundant allele (76.3%; 196/257) in all representatives; the KPC-3 allele was present in 18.3% (47/257). Other KPC variants identified were KPC-4 (n = 7), -5 (n = 1), -6 (n = 1), -10 (n = 1) -12 (n = 3), -17 (n = 1), and -21 (n = 1). We identified 273 KPC genes in all 257 representative plasmids due to KPC colocalizations like KPC-17 and KPC-2 in plasmid pF77_1 (Accession Number CP026137).

Other β-lactamases were present in 55.6% of all representative KPC plasmid sequences (143/257); in some cases, they were localized on integron structures as described previously for OXA-β-lactamases (class D)^30^. All detected β-lactamase combinations for the representatives can be found in the Supplementary Material Table S1, Section 1. The TEM β-lactamases (Ambler class A) (TEM-1 > TEM-150 > TEM-12 > TEM-40) most frequently accompanied the KPC alleles (37.7%; 97/257). TEM-12 is known as an extended spectrum β-lactamase (ESBL)^31^ hydrolysing 3^rd^ generation cephalosporins and TEM-40 as a β-lactamase inhibitor resistant variant^32^. The second most common β-lactamases found in the KPC plasmids were various OXA variants (Ambler class D) (22.6%; 58/257), which do not belong to the carbapenemase-rich ‘*Acinetobacter* cluster’^2^. The identified variants were the narrow-spectrum β-lactamases OXA-1, -9, -10, and the ESBL OXA-129.

Other Ambler class A β-lactamases found less frequently were ESBLs of the CTX-M group (14.0%; 36/257) and SHV variants (14.4%; 37/257), which might exhibit narrow or ESBL phenotypes depending on the variant. Occasionally, β-lactamases such as the GES (ESBL), IMP (Carbapenemase), VEB (ESBL), the CARB or LAP (narrow spectrum β-lactamases), or the Ambler class C cephalosporinases CMY, DHA or FOX were identified.

### Other plasmid features

Most representatives contained various types of genes that explain their successful spread and persistence. For example, in 48.6% *stbB*, *parA* and/or *parM* genes coding for plasmid stability proteins were found, both of which ensure inheritance to daughter cells during cell division. In addition, 38.5% of all representatives utilized a toxin/antitoxin system (usually a type II toxin-antitoxin system such as *ccdA*, *higA*, *maze* or *yefM*), which ensures high plasmid stability within the cells.

The *psiB* gene encoding a plasmid SOS inhibition protein B could be identified in 38.9% of all representatives. The PsiB protein is produced in the recipient during conjugation and suppresses an SOS DNA repair response by inhibiting all activities of the RecA protein^33^. Additionally, most of the plasmid groups (67.3%) contained an *umuDC* operon that encodes the error-prone DNA polymerase. This suggests that the presence of these genes potentially promotes the generation of allelic variants through point mutations for KPCs, but also for the other accompanying β-lactamases.

Presumably, resistance plasmids in general originated from the environment and could still carry typical environmental resistance genes^34,35^. The corresponding genes for mercury resistance (*mer*)^36^, were found in 34.2% (88/257) of all representatives, such as mercury reductase *merA*^37^ or other genes like *merB*, *merC*, *merP*, *merR*, *merT*. Some plasmids also carried resistance genes to arsenic (*ars*) (9.3%; 24/257)^38^ and copper (*cop*) (6.6%; 17/257)^39^. A specific distribution pattern for the heavy-metals resistance genes could not be correlated to the Inc groups or transposons within the analysed KPC plasmids. The exception is the apparent presence of mercury resistance, which is commonly found in environmental samples^40–42^.

### Fusion plasmids or hybrid plasmids

Resistance plasmids have a highly heterogeneous sequence content, and mosaic plasmids consisting of two fused plasmids are not uncommon (e.g. IS26-mediated)^43,44^. As already mentioned, a large number of representatives (29.6%) have several Inc groups.

It is also interesting that in 13 representatives several KPC genes were present. Ten of these plasmids have a duplicate of the same transposon type (nine representatives with a Tn*4401*-like duplicate, one with a Tn*1721A*-like duplicate). This indicates that a transposition event caused these fusion plasmids. One might suggest that these entries are assembler chimeras; however, the plasmid pMNCRE44_5 (Accession Number CP010881.1, representative number 15) was sequenced using high-throughput (Illumina) and long-read sequencing technologies (PacBio) that usually resolves repeat regions commonly found in plasmids. In addition, the results were confirmed via PFGE^45^. During the transposition between the donor and acceptor plasmids guided by Tn*3*, a temporary fusion plasmid is formed in which the transposon is replicated^46^. Usually, this fusion plasmid is resolved by the resolvase. In the case of pMNCRE44_5, the plasmid seems to be not resolved; thus, it consists of two plasmids bearing two KPC-carrying transposons. Plasmid pMNCRE44_5 has a total size of 116,803 bp with two segments: one 56,475 bp segment with IncFIA and another 46,317 bp segment with IncX3. Both segments are connected to each other via Tn*4401*-like elements on both sides. Therefore, new types of KPC resistance plasmids may also arise due to incomplete or incorrect transpositions. However, as we are not aware of the complete assembly methodology of the representatives, we refrain from further speculation, as in some cases assembly chimeras are still likely.

## Conclusions

Plasmid classification, typing or even grouping across different types remains difficult. Most resistance plasmids contain plenty of non-conserved and variable genes or gene cassettes and other genetic elements^10,47^. Gene rearrangements, horizontal gene transfer, and higher mutation rates additionally drive the diversification of plasmids^48^. There are several cgMLST typing schemes based on the specific plasmid backbone of an Inc group (https://pubmlst.org/plasmid/), but these genes are usually not conserved across different types of plasmids^49,50^. Furthermore, identifying the most suitable core backbone remains challenging for certain plasmid types^20^. In addition, essential bacterial genes that would be suitable as a consistent core genome are favourably chromosomally localized^21^. Considering all these limitations when analysing a large number of different plasmids, we applied a cluster-based approach to limit the influence of common plasmids. This should provide a less biased overview of the current distribution of KPC and its various plasmid backbones. This approach was intended and tested for the KPC plasmids and is not universally applicable to other plasmids. The results could show how diverse the KPC carrying plasmids already are: they cover at least 22 known Inc groups and 20.6% of the plasmids could not be assigned to any Inc group. The use of two or more functioning replicons is also widespread, reducing the incompatibility effects to allow for a wider host range^51^. These Inc colocalizations leads to numerous problems when investigating the dynamics and epidemiology of such resistance plasmids. For example, Inc group classification alone for epidemiological purposes would have insufficient resolution or discrimination for multi-replicon plasmids and would not extend to new plasmids either. Long-read sequencing technologies increasingly generate more complete plasmid sequences, but new analysis tools have to be developed which are specifically tailored for the genetic variability of the plasmids. This is where classic ‘backbone’ analyses of core genetic loci are limited^52^.

In total, approximately 1/3 of the analysed representative sequences were either fusion plasmids or hybrid plasmids containing two or more Inc groups. The currently understood modifications of the plasmid backbone via vertical and horizontal gene transfer make it already difficult to trace back the origin of a particular resistance gene^50^, but the potential fusion of plasmids like pMNCRE44_5 (IncFIA and IncX3, Accession CP010881) makes epidemiological investigations even more difficult. The consequence of fused or cointegrated plasmids for public health is difficult to estimate. Assigning a similar trend for other Gram-negative resistance plasmids, PCR-based detection methods may no longer be suitable for trustworthy clinical epidemiology. Whole genome sequencing offers many benefits in this regard but there are still some limitations hampering a comprehensive use in clinical routines or surveillance. For example, the development of automated pipelines is complex and dynamic as more and more bioinformatic tools become available, especially for long-read sequencing technologies. Standardized quality control and data interpretation requires additional bioinformatics know-how and infrastructure that supports data analysis, transfer and long term storage^53^. The general increase in sequencing activity^54^ and different assembly quality might have biased the presented results, but the recent increased use of long-read sequencing technologies and new bioinformatic methods will provide more insights to better understand the overall plasmid dynamics in the future.

Moreover, knowledge of endemic spreading of a particular KPC plasmid might improve empirical therapy, as accessory resistance genes or mechanisms might be localized on the plasmid interfering with the recommended therapy.

## Materials and Methods

### Data extraction and clustering

**Figure 3:**
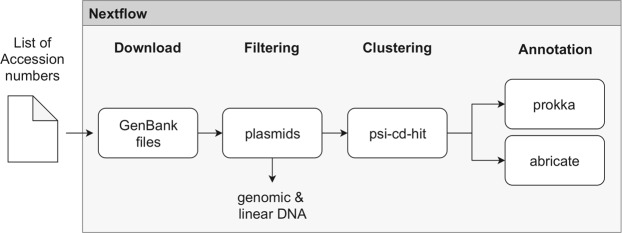
Schematic illustration of the workflow for the clustering and annotation of the KPC-plasmids in Nextflow. Workflow is stored at https://github.com/replikation/plasmid_analysis.

The KPC-2 sequence (Accession Number NC_019161.1) was used for a nucleotide-nucleotide BLAST search in the NCBI database (expected threshold 10E-70), resulting in 759 sequence hits (in May 2019). This list of sequences was analysed by a custom-made workflow (see ‘deposition’ section and Fig. 3). The workflow downloaded the respective GenBank entries from NCBI and filtered, clustered and annotated them. The downloaded gene bank entries were filtered based on their stored information. Only circular entries with a plasmid flag were included for further analysis. The sequences were clustered with psi-cd-hit.pl to identify representative sequences^55,56^. The following settings for psi-cd-hit.pl were used: -aL 0.6 -prog blastn -c 0.6 -g 1 -s “-evalue 10E-100 -max_target_seqs 100000 -qcov_hsp_perc 10 -max_hsps 10”. Only the representative sequence of each cluster (as labelled via psi-cd-hit.pl) was used for further analysis.

### Annotation

The representative sequences were annotated with Prokka^57^ and abricate (https://github.com/tseemann/abricate). Abricate was used with three databases. The NCBI BioProject 3130476 database for the identification of resistance genes. The PlasmidFinder database^24^ for identifying Inc groups and a custom transposon sequence set. Three transposon types were used for identification: Tn*4401* (Accession Number NC021660 and CP004367.2)^58^, Tn*1772A* (Accession Number KF826292)^29^ and Tn*6367*/Tn*6296* (Accession Number MF156709.1 and MF156712)^59^.

Only resistance genes with a coverage of >80% and >75% identity (proportion of exact nucleotide matches) were accepted. All identified β-lactamase genes have an identity of at least 98%. Inc groups have to cover at least 95% with >75% identity in order to be considered. Only transposon hits with a coverage of >60% are considered. All transposon hits have at least 97% identity. The coverage information of each transposon hit is included in Supplementary Material Table S1, Section 1. All representative sequences were analysed with Prokka, so the annotations of all sequences are based on the same settings. The results were used to identify additional plasmid features such as toxin/antitoxin systems or mercury resistance. Where appropriate, isolation dates have been taken from GenBank entries or related publications. Otherwise, the date of submission was used. Metadata such as organism or occurrence country were also extracted (see Supplementary Material Table S1, Section 1).

The β-lactamase genes in all representatives were subsequently confirmed with abricate with the databases NCBI BioProject 3130476, CARD^60^, ARG-ANNOT^61^ and ResFinder^62^ (not part of the workflow). We observed no differences for the group designations (e.g. KPC, TEM, SHV). We only observed a few re-occurring allelic differences for certain TEM or SHV β-lactamases, e.g. TEM-1 (in NCBI), TEM-1A_1 (in ResFinder), TEM-122 (in ARG-ANNOT), TEM-122 (in CARD).

### Plasmid clustering

To cluster the 257 representative KPC plasmids, the distance between each one was determined using ‘Mash V.2.1.1’^25^, which estimates genome distances based on shared k-mer samples using the MinHash algorithm. First, ‘sketches’ were created for the 257 representatives and stored in a ‘sketch database’. Then, each plasmid was compared to the ‘sketch database’ to calculate the distance between each. The calculated distance table was converted into a matrix via R and then plotted with ‘gplots’ (https://cran.r-project.org/web/packages/gplots/index.html).

### Deposition

The whole workflow is illustrated in Fig. 3, written in Nextflow^63^ (www.nextflow.io) and stored at https://github.com/replication/plasmid_analysis. For reproducibility, each step in the workflow runs in a docker container. Each container uses a defined program version, which is stored in a docker image either on dockerhub.com or biocontainers.pro. The metadata of all representative sequences, the cluster results and all 759 Accession Numbers can be found in Supplementary Material Table S1, Sections 1, 2 and 3.

## Supplementary information

Supplementary Material Figure S1

Supplementary Material Table S1
