# Supplementary material for: Assessing genetic diversity and similarity of 435 KPC-carrying plasmids
Source: Sci Rep. 2019 Aug 2;9:11223. doi: 10.1038/s41598-019-47758-5 (PMC6677891; doi:10.1038/s41598-019-47758-5)
Supplement: Supplementary file 1 — Supplementary Material Figure S1 [file 41598_2019_47758_MOESM1_ESM.pdf]

Supplementary Material Figure S1

Assessing genetic diversity and similarity of 435 KPC-carrying plasmids

Christian Brandt (#, 1, 2), Adrian Viehweger (3, 4), Abhijeet Singh (2), Mathias W. Pletz (1, 5), Daniel Wibberg (6), Jörn Kalinowski (6), Sandrina Lerch (1), Bettina Müller (2), Oliwia Makarewicz (1, 5)

---

1 Institute for Infectious Diseases and Infection Control, Jena University Hospital, Jena, Germany

2 BioCenter, Department of Molecular Sciences, Swedish University of Agricultural Sciences, Uppsala, Sweden

3 Institute of Medical Microbiology, University Hospital, Leipzig, Germany

4 Bioinformatics Faculty of Mathematics and Computer Science, Friedrich Schiller University, Jena, Germany

5 InfectoGnostics Research Campus, Jena, Germany

6 Center for Biotechnology, Bielefeld University, Bielefeld, Germany

# Corresponding author

Content: Distance matrix of plasmid sequences.

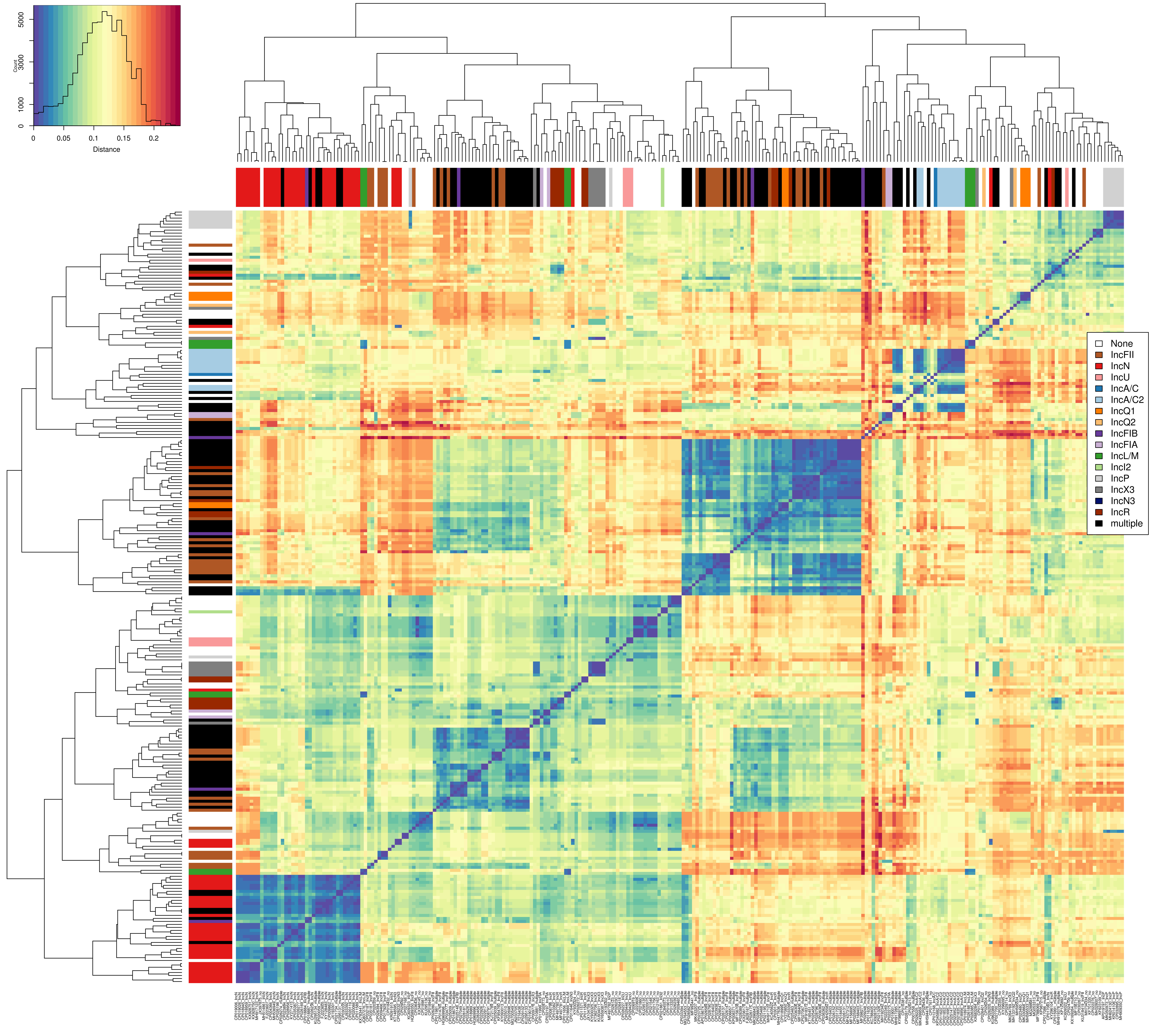
Count
5000
3000
1000
0
0
0.05
0.1
0.15
0.2
Distance
None
IncFII
IncN
IncU
IncA/C
IncA/C2
IncQ1
IncQ2
IncFIB
IncFIA
IncL/M
IncI2
IncP
IncX3
IncN3
IncR
multiple
